# Germline and somatic mosaicism for *FGFR2* mutation in the mother of a child with Crouzon syndrome: Implications for genetic testing in “paternal age-effect” syndromes

**DOI:** 10.1002/ajmg.a.33513

**Published:** 2010-07-15

**Authors:** Anne Goriely, Helen Lord, Jasmine Lim, David Johnson, Tracy Lester, Helen V Firth, Andrew OM Wilkie

**Affiliations:** 1 Weatherall Institute of Molecular Medicine, University of OxfordOxford, UK; 2 Genetics Laboratory, Churchill Hospital, Oxford Radcliffe Hospitals NHS TrustOxford, UK; 3 Craniofacial Unit, John Radcliffe Hospital, Oxford Radcliffe Hospitals NHS TrustOxford, UK; 4 Medical Genetics, Addenbrookes Hospital, Cambridge University Hospitals NHS Foundation TrustCambridge, UK

**Keywords:** craniosynostosis, paternal age-effect disorders, Crouzon syndrome, germline mosaicism, *FGFR2*

## Abstract

Crouzon syndrome is a dominantly inherited disorder characterized by craniosynostosis and facial dysostosis, caused by mutations in the fibroblast growth factor receptor 2 (*FGFR2*) gene; it belongs to a class of disorders that mostly arise as de novo mutations and exhibit a near-exclusive paternal origin of mutation and elevated paternal age (“paternal age effect”). However, even if this is the major mode of origin of mutations in paternal age-effect disorders, germline mosaicism may also occur. Here we describe the first molecularly documented evidence of germline and somatic mosaicism for *FGFR2* mutation, identified in the mother of a child with Crouzon syndrome caused by a heterozygous c.1007A>G (p.Asp336Gly) substitution. Levels of maternal somatic mosaicism for this mutation, estimated by pyrosequencing, ranged from 3.3% in hair roots to 14.1% in blood. Our observation underlines the importance of parental molecular testing for accurate genetic counseling of the risk of recurrence for Crouzon, and other paternal age-effect syndromes. © 2010 Wiley-Liss, Inc.

## INTRODUCTION

Crouzon syndrome (OMIM #123500) comprises craniosynostosis, exorbitism, hypertelorism, midface hypoplasia, hooked nose, thin vermilion of the upper lip and mandibular prognathism, leading to dental malocclusion. Although the limbs are traditionally described as normal, subtle alterations, for example in metacarpophalangeal proportions of the hands, are a consistent finding [Murdoch-Kinch and Ward, 1997]. Crouzon syndrome is caused by over 40 different heterozygous missense mutations in the fibroblast growth factor receptor 2 (*FGFR2*) gene [reviewed in Passos-Bueno et al., 2008]. Although Crouzon syndrome is inherited as an autosomal dominant trait, many cases are sporadic and present as de novo mutations arising from unaffected parents. Such de novo mutations were found to arise exclusively on the father's allele (n = 11) and to exhibit a paternal age effect (increased average age of the father compared to the population mean) [Glaser et al., 2000]. Similar observations have been made for other disorders involving dominant mutations in *FGFR2* or *FGFR3*-including Apert [Moloney et al., 1996], Pfeiffer [Glaser et al., 2000], and Muenke [Rannan-Eliya et al., 2004] syndromes and achondroplasia [Wilkin et al., 1998].

There is now good evidence that this class of so-called “paternal age-effect” mutations originate as rare spontaneous events within spermatogonial cells of adult males. Because they confer a selective advantage to the mutant stem or progenitor cells, these mutations are progressively enriched over time by a process of clonal expansion, resulting in elevated levels of mutation in sperm [Goriely et al., 2009]. Although it is well documented that the relative risk increases with advancing paternal age, there is nevertheless a very low absolute probability of recurrence within a family, usually estimated to be well under 1% [Mettler and Fraser, 2000].

Apparently at variance with this usual mechanism of mutation in paternal age-effect syndromes, several instances of germline mosaicism in Crouzon syndrome have been suggested [Rollnick, 1988; Kreiborg and Cohen, 1990; Navarrete et al., 1991]. However, all these reports predated the identification of *FGFR2* mutations in Crouzon syndrome [Reardon et al., 1994] and none has been molecularly confirmed. Here, we present the first molecularly proven case of germline and somatic mosaicism for *FGFR2* mutation and discuss the implication of this finding in the context of recurrence risk estimation for paternal age-effect disorders as a whole.

## MATERIALS AND METHODS

### Identification of *FGFR2* Mutation

The study was approved by the Oxfordshire Research Ethics Committee B (C02.143) and written informed consent was obtained from the parents. Genomic DNA was extracted from saliva using the OrageneDNA OG-250 kit (DNA Genotek, Kanata, Ontario, Canada) following the manufacturer's instructions, and from peripheral blood and/or hair roots by proteinase K digestion and phenol/chloroform extraction. Nomenclature for *FGFR2* (IIIc spliceform) follows the cDNA reference sequence (Genbank NM_000141.4, where the numbering of the cDNA nucleotide starts with +1 at the A of the ATG initiation codon). PCR and sequencing of *FGFR2* exons containing hotspots of mutations for craniosynostosis syndromes (exons 8 [IIIa] and 10 [IIIc]; nomenclature according to Kan et al. 2002) was initially performed in a clinical diagnostic laboratory; mutations in exon IIIa were excluded. For further sequence analysis, amplification of exon 10 (IIIc) was performed in a 30 µl PCR reaction using Expand High Fidelity^PLUS^ PCR system reagents (Roche Applied Biosystems, Mannheim, Germany) in the following conditions: 1× Hifi^PLUS^ buffer, 2.5 mM MgCl_2_, 0.75 U Hifi^PLUS^ DNA polymerase, 200 µM dNTPs and 0.1 µM each primer (5′-CCTCCACAATCATTCCTGTGTC-3′ and 5′-ATAGCAGTCAACCAAGAAAAGGG-3′). The following cycling conditions were used: initial 94°C for 2 min, followed by 30 cycles of 94°C for 10 sec, 62°C for 30 sec, 72°C for 30 sec, followed by a final extension at 72°C for 10 min. After checking for amplification on a 2% agarose gel, the fragments were gel-purified using a gel extraction microelute column (EZNA, Omega Bio-Tek, Norcross, GA). The amplicons were then subjected to sequencing in both orientations using the PCR primers and fluorescently labeled dideoxy-terminator reactions and run on an ABI 3700 automated DNA sequencer (Applied Biosystems, Carlsbad, CA).

Restriction digest with *Hga*I (New England Biolabs, Ipswich, CA) was performed on 15 µl of the pyrosequencing PCR product (see below) in a 30 µl reaction containing 1× Buffer 1 and 5 U of *Hga*I for 4 hr at 37°C. The resulting digest was run on a 4% TBE agarose gel.

Microsatellite analysis (*D10S1483*) was performed after amplification with the primers 5′-FAM-CAATGCTATCCCGGCTATG-3′ and 5′-TCAAGACTGCAAGCGTGT-3′ in Invitrogen (Carlsbad, CA) PCR reagents (1× PCR buffer, 2.5 mM MgCl_2_, 1 U TAQ DNA polymerase, 200 µM dNTPs, 0.1 µM each primer and 20–50 ng genomic DNA) with the cycling conditions: initial 94°C for 2 min, followed by 35 cycles of 94°C for 15 sec, 56°C for 30 sec, and 72°C for 30 sec. The DNA fragment analysis was performed on ABI 3730 DNA Analyzer and scored using the GeneMapper software (Applied Biosystems).

### Pyrosequencing

The following pyrosequencing primers were designed to amplify a 132 bp *FGFR2* exon IIIc fragment: 5′-TAACACCACGGACAAAGAGATTGAGGTTCTC-3′ and 5′-Biotin-GGCAGAACTGTCAACCATGCAGAGTGAA-3′ using High Fidelity PCR system reagents (Roche Applied Biosystems) in a 60 µl PCR reaction containing 1× Hifi buffer, 1.5 mM MgCl_2_, 0.5 U Hifi DNA polymerase, 200 µM dNTPs, 0.1 µM each primer and 20–50 ng genomic DNA with the cycling conditions: initial 94°C for 2 min, followed by 35 cycles of 94°C for 10 sec, 62°C for 30 sec, 68°C for 30 sec, followed by a final extension at 68°C for 10 min. This PCR reaction was performed three times independently and after checking for amplification on a 2% agarose gel, 10 µl of each PCR product was pyrosequenced twice on a PyroMark Q96 MD (Qiagen, Hilden, Germany) using the sequencing primer 5′-CGGAATGTAACTTTTG-3′ and following a protocol previously described [Twigg et al., 2006]. After dispensation of the enzyme (E) and substrate (S), the nucleotides were dispensed in the order A-C-G-C-G-T-C-A-G-C-G-T-C-T. This order generates four peaks specific for the normal c.1007A allele (dispensations C_10_, G_11_, C_13_, and T_14_) and four mutant-specific peaks (C_4_, G_5_, C_7_, and T_12_), which were averaged to estimate the relative levels of c.1007G mutant allele. Analysis of the peak heights follows the same principles described in Twigg et al. 2006.

## RESULTS

**FIG. 1:**
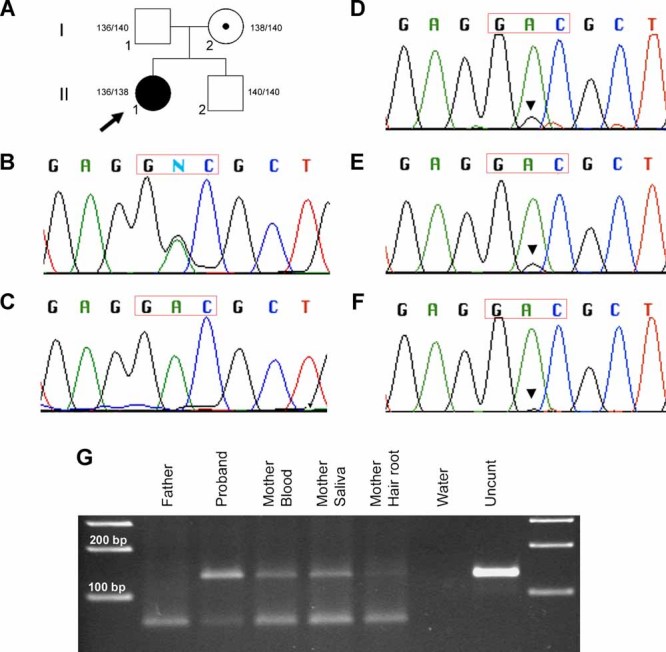
*FGFR2* exon IIIc sequence and restriction enzyme digestion **A**: Pedigree indicating the segregation of the microsatellite *D10S1483* located 6.7 kb away from the site of the *FGFR2* mutation in the proposita (II-1), showing that the two sibs have inherited opposite maternal *FGFR2* alleles. B–F: DNA sequencing chromatograms around the mutation site (p.Asp336 codon corresponding to the GAC boxed sequence) in *FGFR2* exon IIIc (**B**,**C**: reverse complementary sequence of the minus strand; **D**–**F**: forward sequence of the plus strand); the proposita's blood DNA (B) shows a heterozygous c.1007A>G mutation encoding a p.Asp336Gly substitution; (C) the same change is absent from the father's blood DNA. D–F: Chromatograms from the mother's blood (D), saliva (E) and hair roots (F) genomic DNA, revealing a variable amount of the c.1007A>G mutation in these tissues (arrowheads). **G**: *Hga*I restriction digest of a 132 bp PCR product for the different genomic DNA samples (as indicated), shows that the normal allele (c.1007A), is cut into two fragments (65 and 67 bp), while the c.1007A>G mutation abolishes the *Hga*I restriction site (GACGCN_5_). Comparison of the ratio of mutant (upper undigested fragment) to normal (lower fragments) alleles in the maternal samples to that of the heterozygous proposita, shows that the relative amount of undigested fragment is reduced in the mother's blood and saliva samples and is barely visible in the hair root sample (right and left lanes are 100 bp ladders). [Color figure can be viewed in the online issue, which is available at http://www.interscience.wiley.com.]

**FIG. 2:**
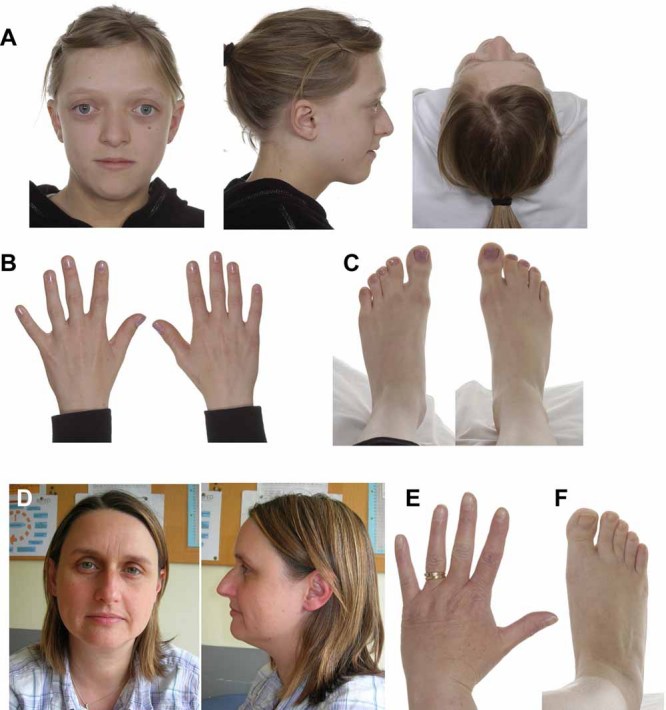
Phenotype of the proposita and her mother. **A**–**C**: Proposita (II-1), age 12 years. A: Facial appearance of the patient at the time of diagnosis. Note hypertelorism, exorbitism, midface hypoplasia, frontal bossing, and mild prognathism. B: Hands of the proposita showing mild 2/3 cutaneous syndactyly and normal thumbs. C: Proposita's feet showing normal big toes and bilateral syndactyly of second and third toes. D,E: Mother of the proposita (I-2), age 40 years. **D**: Her face is normal and she does not display any crouzonoid features. **E**,**F**: Her hands and feet appear normal. [Color figure can be viewed in the online issue, which is available at http://www.interscience.wiley.com.]

The proposita II-1, the first of two children born to healthy, nonconsanguineous north-European parents (Fig. 1A), presented at the age of 12 years with recurrent otitis media, conductive hearing loss and visual disturbance. Grommets (tympanostomy tubes) had been inserted at age 5 years and spectacles prescribed at 12 years. Exorbitism was noted at the time of the ophthalmological examination and she was referred for a clinical genetics opinion at which time it was noticed that she had midface hypoplasia, ocular proptosis, hypertelorism, frontal bossing, short upper lip, mild prognathism (Fig. 2A), a high narrow palate, dental crowding, mild 2/3 cutaneous syndactyly of her hands (Fig. 2B) and bilateral syndactyly of the second and third toes (Fig. 2C). No intellectual or developmental impairment was apparent and there was no significant family history. 3D-computed tomography of the skull showed pan-synostosis and absence of sutures as well as reduced space with copper beating suggestive of raised intra-cranial pressure (ICP). However, direct measurement of ICP was normal, and she is being managed conservatively pending a detailed neuropsychological assessment. The clinical features fall within the overlap zone between Pfeiffer and Crouzon syndromes, which are also known to overlap at the molecular level [Rutland et al., 1995; Kan et al., 2002]; Crouzon syndrome was favored as the final diagnosis because of the late presentation and the mild craniofacial phenotype.

Analysis of mutation hotspots in *FGFR2* (exons IIIa and IIIc) performed on blood genomic DNA showed a heterozygous c.1007A>G mutation, encoding a p.Asp336Gly substitution within the third immunoglobulin-like domain of FGFR2 (Fig. 1B). This mutation has been reported once previously, also in a patient diagnosed with Crouzon syndrome [Stenirri et al., 2007].

Subsequently, genetic testing of peripheral blood from the patient's parents was requested. The father's *FGFR2* sequence was normal (Fig. 1C), but the sample from the mother (I-2), 28.5 years at the time of the girl's birth, was found to be mosaic for the same c.1007A>G mutation (Fig. 1D). She appeared to be clinically normal (Fig. 2D–F), and her skin (including examination under a Wood's lamp) was normal. Further maternal samples, including hair roots and saliva were obtained and tested. Direct sequencing showed that the mutation could be detected at variable levels in all tissues (Fig. 1E,F). This result was further verified using a diagnostic restriction digest (Fig. 1G). The *FGFR2* mutation was not present in a mouth-brushing sample from the patient's clinically normal younger brother (II-2) (data not shown); based on the segregation of a polymorphic (CA)_n_ microsatellite (*D10S1483*) located 6.7 kb 5′ of the *FGFR2* mutation site, he inherited the opposite maternal chromosome to his affected sister (Fig. 1A).

**FIG. 3:**
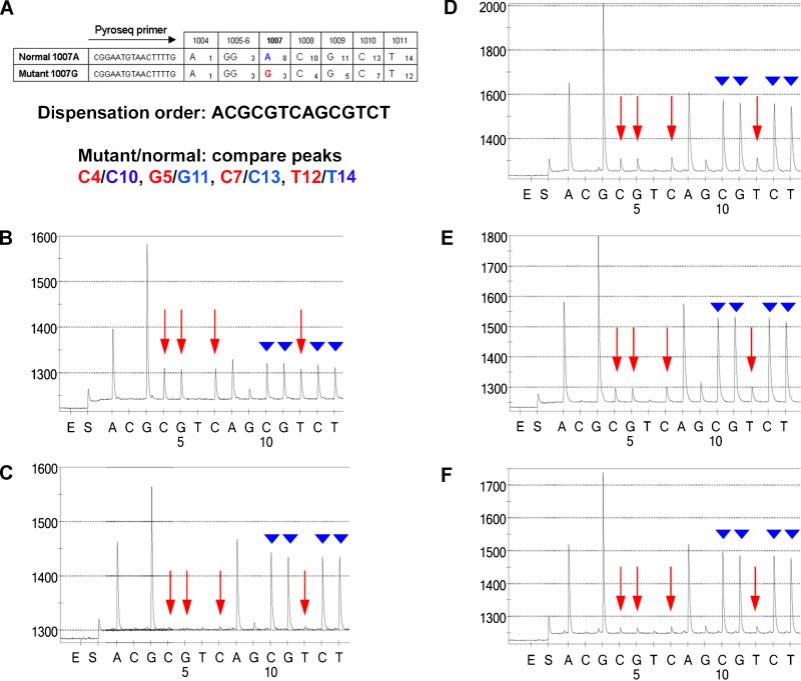
Pyrosequencing and quantification of the mosaicism levels for the *FGFR2* c.1007A>G mutation. **A**: Pyrosequencing scheme showing a table with the sequence context of the 2 *FGFR2* alleles (normal c.1007A (above) and mutant c.1007G (below)) and the sequencing primer (Pyroseq primer). The allele-specific incorporation of nucleotides is listed for each dispensation (denoted by the numbers on the right side in each box). Below, the dispensation order in which the nucleotides are added is indicated. This generates four mutant-specific peaks (at the 4th, 5th, 7th, and 12th dispensations) and four peaks unique for the normal allele (the 10th, 11th, 13th, and 14th dispensations). **B**–**F**: Typical pyrograms showing the four mutant-specific peaks (red arrows) and the normal allele-specific peaks (blue arrowheads) for the proposita's blood DNA (B), the father's blood DNA (C), the mother's blood DNA (D), mother's saliva DNA (E) and mother's hair root DNA (F). [Color figure can be viewed in the online issue, which is available at http://www.interscience.wiley.com.]

Pyrosequencing was used to estimate the levels of mosaicism more precisely in the three different maternal tissues. The assay was designed to compare four peaks generated specifically to the normal allele with four mutant-specific peaks (Fig. 3A). The assay was validated by measuring the levels of mutant allele in the heterozygous proposita DNA, where the *FGFR2* c.1007A>G mutation was estimated to be at a level of 48.9% (±0.4%) (Fig. 3B), and in the normal control paternal DNA, where it was found to be at a background level of 1.2% (±0.4%) (Fig. 3C). After adjustment of the raw measurements by linear interpolation, we confirmed the mosaicism in the maternal samples and estimated the proportion of the c.1007G allele to be 14.1% (±0.6%) in blood (Fig. 3D), 13.3% (±0.4%) in saliva (Fig. 3E) and 3.3% (±1.1%) in hair roots (Fig. 3F).

## DISCUSSION

We report on the first molecularly proven case of a somatic and germline mosaicism for a dominant mutation in the *FGFR2* gene. The heterozygous c.1007A>G (p.Asp336Gly) substitution appears to cause a fairly mild phenotype, as the proposita was diagnosed with Crouzon syndrome only at the age of 12 years and has not yet required surgery. A similar presentation of postnatal craniosynostosis and increased ICP in Crouzon syndrome was described previously [Connolly et al., 2004; Hoefkens et al., 2004] and although ICP measurements were normal in the proposita, she will continue regular follow-up to monitor for any symptomatic changes suggestive of increased ICP.

Crouzon syndrome belongs to a group of genetic disorders that have been termed “paternal age-effect” disorders to reflect the fact that the dominantly acting causative mutations, when they arise de novo, exhibit a near-exclusive paternal origin and an increased paternal age [Crow, 2000; reviewed in Goriely et al., 2009]. As well as mutations in *FGFR2* and *FGFR3*, mutations in *RET* (causing multiple endocrine neoplasia types 2A and 2B), *PTPN11* (causing Noonan syndrome) and *HRAS* (causing Costello syndrome) also belong to this group [reviewed in Goriely et al., 2009]. Recently, it was proposed that the causative mutations responsible for the paternal age-effect disorders originate by a shared mechanism taking place during spermatogenesis. These mutations arise as rare events in the testes of most or all men, during the repeated mitotic replications of the spermatogonial stem or progenitor cells required for the production of mature sperm. Because they encode dominant gain-of-function proteins, the paternal age-effect mutations confer a selective advantage to the spermatogonial cells in which they originate, leading to clonal expansion of the mutant stem cells over the course of time [Goriely et al., 2003, 2005, 2009]. Although the resulting enrichment of sperm carrying a paternal age-effect mutation could formally be considered “germline mosaicism,” direct measurements of mutation levels in sperm have shown that these do not exceed 1 in 1,000 [Goriely et al., 2003, 2005, 2009; Choi et al., 2008; Yoon et al., 2009]. For this reason, a healthy couple with a single child with one of the paternal age-effect disorders will generally be counseled as having a low recurrence risk (below 1%). However, it is important to distinguish this from the classical process of germline mosaicism, which can occur in both males and females, originates in the early embryo rather than in the adult and as a result, carries a risk of recurrence in offspring that is several orders of magnitude higher (up to 1 in 2).

As exemplified in this report, even if most new cases of paternal age-effect syndromes arise as de novo mutations in the paternal germline, they can also occur by a classical mechanism of parental mosaicism. Whilst this is an unusual situation, it is not unique to *FGFR2* and similar cases have been documented for *FGFR3* mutations in achondroplasia [Henderson et al., 2000; Natacci et al., 2008] and in thanatophoric dysplasia [Hyland et al., 2003], and for *HRAS* mutations in Costello syndrome [Gripp et al., 2006; Sol-Church et al., 2009]. Generally these cases came to attention after it was noticed either that (1) more than one pregnancy was affected in a sibship or (2) one of the parents showed partial manifestation of the disorder. In the present report, despite the fact that neither of these criteria was present, the sequence analysis of the DNA of the patient's parents showed that the mother, who is phenotypically normal, is mosaic for the *FGFR2* c.1007A>G mutation carried by her affected daughter. As this is a heterozygous mutation, we estimated that 25–30% of her blood and saliva cells and less than 10% of her hair root cells carry the mutation. Since her daughter has inherited the *FGFR2* c.1007A>G substitution as a germline mutation, it must be present in the mother's germ cells as well. Therefore, the mother presents with somatic and germline mosaicism. As we established that her unaffected son inherited the opposite maternal allele to his sister's, it remains possible that a large proportion of the mother's germ cells carry the pathogenic c.1007A>G mutation, leading to an offspring recurrence risk up to 50%. Although *FGFR2* somatic mosaicism—causing acneiform naevus in the epidermis—has been documented previously [Munro and Wilkie, 1998; Melnik et al., 2008], to our knowledge, the present report is the first case of molecularly proven germline and somatic mosaicism for a *FGFR2* mutation.

In conclusion, this report underlines the importance of checking for parental mosaicism even in apparently de novo paternal age effect syndromes. With current DNA sequencing technology, detection of somatic mosaicism at levels as low as 10% should be routinely possible and counseling of recurrence risk for paternal age-effect syndromes for a family with a single affected child improved.
